# Smart and Future Applications of Internet of Multimedia Things (IoMT) Using Big Data Analytics

**DOI:** 10.3390/s22114146

**Published:** 2022-05-30

**Authors:** Rohit Sharma, Damianos Gavalas, Sheng-Lung Peng

**Affiliations:** 1 SRM Institute of Science and Technology, Ghaziabad 201204, India; 2 Department of Product and Systems Design Engineering, University of the Aegean, 84100 Syros, Greece; dgavalas@aegean.gr; 3 Department of Creative Technologies and Product Design, National Taipei University of Business, Taipei 100, Taiwan; slpeng@ntub.edu.tw

This Special Issue is focused on breakthrough developments in the field of Internet of Multimedia Things (IoMT), particularly on smart and future applications of IoMT using big data analytics. The selected contributions report current scientific progress in a wide range of topics covering security challenges and protection methods in IoMT with an emphasis on healthcare applications; specific topics include user behavior aspects of mobile vs. non-mobile users of Internet video streaming services and IoT-enabled statistical and machine learning techniques to recognize cardiac disease features related to heart failure. This Special Issue of the MDPI journal *Sensors* on ‘‘Smart and Future Applications of Internet of Multimedia Things (IoMT) Using Big Data Analytics” solicited high-quality research on recent advances related to the intelligent Internet of Multimedia Things (IoMT) and big data analytics. Contributions tackled open research problems, integrated efficient novel solutions, carried out performance evaluation studies, and compared state-of-the-art solutions. The Special Issue attracted numerous submissions. Following a rigorous review process, three outstanding papers were finally selected for inclusion in the Special Issue. Each paper was reviewed by three independent experts. The accepted papers cover a wide spectrum of research topics in the broader area of the Special Issue.

The first paper [1], entitled “Internet of Things: Evolution, Concerns and Security Challenges”, was co-authored by P. Malhotra, Y. Singh, P. Anand, D.K. Bangotra, P.K. Singh, and W.-C. Hong. The article comprises a survey of IoT technologies with emphasis on security challenges, particularly on various attacks and anomalies, as well as existing detection methods. The survey provides in-depth analysis and assessment of diverse machine learning and a deep learning-based network intrusion detection system (NIDS). The article also presents a case study of healthcare in IoT. The study depicts the architecture, security, and privacy issues and application of learning paradigms in this sector. Last, the paper offers insights on research challenges in this field of research to allow further rectifications in existing approaches to deal with unusual complications.

The second paper [2], entitled “Mobile vs. Non-Mobile Live-Streaming: A Comparative Analysis of Users Engagement and Interruption Using Big Data from a Large CDN Perspective”, was co-authored by D.V.C. da Silva, A.A. de A. Rocha, and P.B. Velloso. The article compares user behavior aspects of mobile vs. non-mobile users consuming and interacting with Internet video streaming services. The research work focuses on metrics such as engagement, interruption, churn, and payload. The article reports on mobile users considering aspects such as the operating system, geolocation, network access, interruption, and engagement. The authors argue that their research results offer insights to improve streaming services. As a case study, the authors used a major streaming service in Brazil and mainly focused on large-scale lives.

The third paper [3], entitled “Cardiac Diagnostic Feature and Demographic Identification (CDF-DI): An IoT Enabled Healthcare Framework Using Machine Learning”, was co-authored by D. Kumar, C. Verma, S. Dahiya, P. Kumar Singh, M.S. Raboaca, Z. Illés, and B. Bakariya. The article presents a public key infrastructure (PKI)-secured IoT-enabled framework entitled the Cardiac Diagnostic Feature and Demographic Identification (CDF-DI) system, which employs statistical and machine learning techniques to recognize several cardiac disease features related to heart failure (HF). Among the many algorithms and models investigated, the random forest (RF) algorithm demonstrated the highest accuracy in determining the survival status of the patient by examining five key features. Additionally, the RF algorithm was the most prominent solution in recognizing the patient’s gender and age group. The authors argue that the recommended features would benefit the clinical practice and would support existing medical support systems in identifying the possibility of the survival status of heart patients.

## Data Availability

Not applicable.
